# A New Nomogram Model for Predicting 1-Year All-Cause Mortality After Hip Arthroplasty in Nonagenarians With Hip Fractures: A 20-Year Period Retrospective Cohort Study

**DOI:** 10.3389/fsurg.2022.926745

**Published:** 2022-06-28

**Authors:** Xingchen Lu, Ziming Wang, Feifei Chong, Yu Wang, Siyu Wu, Quanyin Du, Wenlong Gou, Keyun Peng, Yan Xiong

**Affiliations:** ^1^ Department of Orthopaedics, Daping Hospital, Army Medical University (Third Military Medical University), Chongqing, China; ^2^ Department of Clinical Nutrition, Daping Hospital, Army Medical University (Third Military Medical University), Chongqing, China

**Keywords:** nomogram, nonagenarians, hip fractures, hip arthroplasty, all-cause mortality

## Abstract

**Background:**

China has become an ageing society and as it continues to age, it will face an increasing number of hip fractures in nonagenarians. However, few preoperative assessment tools to determine the postoperative mortality risk in nonagenarians with hip fracture were available. The aim of this study was to identify all-cause mortality risk factors after hip arthroplasty in nonagenarians with hip fractures and to establish a new nomogram model to optimize the individualized hip arthroplasty in nonagenarians with hip fractures.

**Methods:**

We retrospectively studied 246 consecutive nonagenarians diagnosed with hip fracture from August 2002 to February 2021 at our center. During the follow-up, 203 nonagenarians with a median age of 91.9 years treated with hip arthroplasty were included, of which 136 were females and 67 were males, and 43 nonagenarians were excluded (40 underwent internal fixation and 3 were lost to follow-up). The full cohort was randomly divided into training (50%) and validation (50%) sets. The potential predictive factors for 1-year all-cause mortality after hip arthroplasty were assessed by univariate and multivariate COX proportional hazards regression on the training set, and then, a new nomogram model was established and evaluated by concordance index (C-index) and calibration curves.

**Results:**

After analyzing 44 perioperative variables including demographic characteristics, vital signs, surgical data, laboratory tests, we identified that age-adjusted Charlson Comorbidity Index (aCCI) (*p *= 0.042), American Society of Anesthesiologists (ASA) classification (*p *= 0.007), Urea (*p *= 0.028), serum Ca^2+^ (*p *= 0.011), postoperative hemoglobin (*p *= 0.024) were significant predictors for 1-year all-cause mortality after hip arthroplasty in the training set. The nomogram showed a robust discrimination, with a C-index of 0.71 (95%CIs, 0.68–0.78). The calibration curves for 1-year all-cause mortality showed optimal agreement between the probability as predicted by the nomogram and the actual probability in training and validation sets.

**Conclusion:**

A novel nomogram model integrating 5 independent predictive variables were established and validated. It can effectively predict 1-year all-cause mortality after hip arthroplasty in nonagenarians with hip fracture and lead to a more optimized and rational therapeutic choice.

## Introduction

The aging process of China is accelerating, 115 million will be over 80 years old and 0.6 million are expected to become centenarians by 2050 (1). Population aged more than 90 years in China accounted for 15% of the global nonagenarians at the end of 2017 (2). The incidence of hip fracture increases with age (3). Unless the patients face high risk of intraoperative death or difficulties to access to surgical care, operative treatment for most hip fractures is recommended, and arthroplasty is generally preferred for the management of displaced femoral-neck fractures and unstable intertrochanteric fractures in the elderly (4, 5), because of the obvious advantages such as early weight-bearing training, reduced bed-related and implant-related complications.

Despite advances in medical and surgical treatments for geriatrics with hip fractures, postoperative mortality has increased with increasing age, especially in nonagenarians with significant comorbidity (6, 7). Several studies have investigated the risk factors contributing to mortality in the extremely old patients (8, 9), with the goal of helping clinicians and surgeons to reduce risk and improve outcomes. Nevertheless, risk factors related to 1-year mortality were rarely investigated in previous studies and few attempts have been made to quantify the contribution of established risk factors to postoperative mortality in nonagenarians with hip fracture. The aim of the present study was to develop a new nomogram model for the management of hip fractures in nonagenarians and to investigate the potential risk factors correlating with 1-year all-cause mortality after hip arthroplasty in nonagenarians.

## Materials and Methods

### Date Source

We performed a retrospective cohort study to assess mortality and the risk factors that influence it following hip arthroplasty in the nonagenarians with hip fractures from August 2002 to February 2021 in Daping Hospital of Third Military Medical University (Army Medical University). The inclusion criteria were as follows: (1) patients aged more than 90 years old; (2) low-energy or fragility-type hip fractures; (3) patients who received total hip arthroplasty or hemiarthroplasty; and (4) availability of complete clinical and follow-up data. Nonagenarians with neoplastic-related pathological hip fractures and multiple fractures, as well as non-operatively managed hip fractures were excluded from the current analysis. The study protocol was approved by the Ethics Committee of Daping Hospital of Army Medical University (2022[89]). All authors had access to the results of the study, reviewed and edited the final manuscript, and were involved in the decision to submit the manuscript for publication.

### Outcome Variables

The primary outcome for this study was mortality, which was obtained from the electronic medical record (EMR) and from telephone follow-ups using a uniformed questionnaire by two trained physicians. The date of the surgery was used as the starting point of the patient’s observed survival time, and the end of follow-up or the date of death was used as the observation end point. The follow-up period ended on February 28, 2022. Patients were considered lost to follow-up if they were out of contact or refused to provide the current information. In order to analyze which variables were the potential risk factors relating to postoperative mortality, 44 preoperative, intraoperative, and postoperative variables were collected and analyzed. Age-adjusted Charlson Comorbidity Index (aCCI) and ASA classification were subsequently calculated for each patient as a global measure of health status (Table 2).

### Model Construction and Evaluation

The patients were randomly assigned into training (50%) and validation (50%) sets. Univariate and multivariate COX proportional hazards regression were performed on the training set to identify the significant independent factors. A nomogram was then established to present these factors and their corresponding points for the risk of 1-year all-cause mortality. The predictive accuracy and discriminative ability were determined using the C-index. Calibration of the nomogram for 1-year all-cause mortality was performed by comparing the predicted and observed probability.

### Statistical Analysis

Statistical analyses were performed using R package version 4.1.0 (Institute for Statistics and Mathematics, Vienna, Austria). Categorical variables were described as count (%) and compared using the chi-square test or Fisher’s exact test, while continuous variables were described as $\bar{x}\pm s$ and compared using the t-test. The missing values of all potential predictors (missing rate of less than 10.0%) were imputed by expectation-maximization (EM) method. Univariate Cox regression analysis was adopted to identify the potential predictive factors which reached a *p* value of less than 0.05 in training set. Then the variables with *p < *0.05 were entered into multivariate COX proportional hazards regression to screen the significant independent factors. Hazard ratio (HR) was estimated, and the corresponding 95% confidence interval (95% CI) and *p* value were reported at the same time. The rms package was used to formulate a prognostic nomogram based on the results of multivariate analysis. The C-index and the calibration curve were applied to assess the discrimination and calibration of the nomogram. *p *< 0.05 was considered statistically significant.

## Results

### Baseline Characteristics

During the study period, 246 nonagenarians who suffered hip fractures underwent operations. Of these, 203 patients who met the inclusion criteria were enrolled, and the remaining 43 were excluded (40 underwent internal fixation and 3 were lost to follow-up). For the 203 nonagenarians, the observed survival time was 1–120 months, average of 31.08 months, median of 60.5 months. The cumulative deaths in postoperative 1 month, 3 months, 6 months and 12 months was 21(10.3%), 30(14.8%), 37(18.2%) and 58(29.6%), respectively. The main causes of death were presented in Table 1. 203 eligible patients were randomly divided into training (50%, *n* = 102) and validation (50%, *n* = 101) sets. The baseline characteristics of the patients including demographic characteristics, vital signs, surgical data and laboratory tests are listed in Table 2. The baseline data were similar between the training and validation set, and deaths of 30 (29.4%) and 28 (27.7%) in the training and validation set, respectively (*p* = 0.790). Preoperative length of stay and serum K+ differed significantly between the training and validation set (*p *< 0.05), while the remaining variables between the two sets presented no difference.

**Table 1:** The main causes of 58 deaths.

System	Disease	Number
MODS	Two or more organ systems presented dysfunctional	13
Cardiogenic Disease	Myocardial Infarction, Cardiac Failure, Malignant Arhythmia	8
Cerebral Diseases	Cerebral Infarction, Cerebral Hemorrhage	5
Respiratory Diseases	Respiratory Failure, Pulmonary Infection	4
Digestive Diseases	Gastrointestinal Hemorrhage, Infection of Digestive canal	2
Accidents	The patients fell while walking and died after a period of time in bed	2
Malignant Tumor	liver cancer	1
Dysoemia	The patients died at home and the causes of death were not confirmed by doctors	23

*MODS, Multiple Organ Dysfunction Syndrome.*

**Table 2:** Demographic and clinical characteristics of the included nonagenarians with hip fracture.

Variables	Training (*n* = 102)	Validation (*n* = 101)	*p*-value
Age cohorts (years), No. (%)			
90–94	89 (87.3)	86 (85.1)	0.663
≥95	13 (12.7)	15 (14.9)	
Gender (female), No. (%)	72 (70.6)	64 (63.4)	0.298
Length before admission (hours), No. (%)			
≤72	46 (45.1)	46 (45.5)	0.736
73–168	34 (33.3)	39 (38.6)	
169–504	16 (15.7)	12 (11.9)	
>504	6 (5.9)	4 (4.0)	
Preoperative length of stay (days), No. (%)			
<2	47 (46.1)	32 (31.7)	0.029
2–3	16 (15.7)	30 (29.7)	
>3	39 (38.2)	39 (38.6)	
Fracture type, No. (%)			
Femoral neck fracture	37 (36.3)	48 (47.5)	0.119
Intertrochanteric fracture	65 (63.7)	53 (52.5)	
Left/Right, No. (%)			
Left	60 (58.8)	60 (59.4)	1.000
Right	42 (41.2)	41 (40.6)	
aCCI, mean (SD)	5.09 ± 1.05	5.03 ± 1.01	0.687
Pulse (beats/min), No. (%)			
<60	4 (3.9)	3 (3.0)	0.418
60–100	89 (87.3)	83 (82.2)	
>100	9 (8.8)	15 (14.9)	
Systolic pressure (mmHg), No. (%)			
90–119	9 (8.8)	18 (17.8)	0.188
120–139	34 (33.3)	38 (37.6)	
140–159	30 (29.4)	24 (23.8)	
160–179	23 (22.5)	14 (13.9)	
≥180	6 (5.9)	7 (6.9)	
Diastolic pressure (mmHg), No. (%)			
<60	8 (7.8)	9 (8.9)	0.596
60–79	54 (52.9)	57 (56.4)	
80–89	32 (31.4)	23 (22.8)	
90–99	7 (6.9)	9 (8.9)	
100–109	1 (1.0)	3 (3.0)	
Cardiac function classification, No. (%)			
NYHA I&II	73 (71.5)	68 (67.3)	0.512
NYHA III&IV	29 (28.4)	33 (32.7)	
ASA classification, No. (%)			
ASA I&II	46 (45.1)	41 (40.6)	0.571
ASA III&IV	56 (54.9)	60 (59.4)	
Operation type, No. (%)			
Hemiarthroplasty	94 (92.2)	97 (96.0)	0.373
Total hip arthroplasty	8 (7.8)	4 (4.0)	
Anesthesia, No. (%)			
Nerve block anesthesia	88 (86.3)	88 (87.1)	1.000
Intravertebral anesthesia	10 (9.8)	9 (8.9)	
General anesthesia	4 (3.9)	4 (4.0)	
Operation time (min), No. (%)			
<60	8 (7.8)	4 (4.0)	0.399
60–120	86 (84.3)	91 (90.1)	
>120	8 (7.8)	6 (5.9)	
Blood transfusion, No. (%)	25 (24.5)	20 (19.8)	0.500
White-cell count (×10^9^/L), No. (%)			
<4.00	2 (2.0)	3 (3.0)	0.879
4.00–10.00	75 (73.5)	72 (71.3)	
≥10.01	25 (24.5)	26 (25.7)	
Platelet count (×10^9^/L), No. (%)			
<100	11 (10.8)	17 (16.8)	0.421
100–300	82 (80.4)	74 (73.3)	
>300	9 (8.8)	10 (9.9)	
Percentage of neutrophl >75%, No. (%)	55 (53.9)	66 (65.3)	0.116
Hematocrit, No. (%)			
≥40 (Male), ≥35 (Female)	21 (20.6)	20 (19.8)	0.889
<40 (Male), <35 (Female)	81 (79.4)	81 (80.2)	
Albumin <35 g/L, No. (%)	65 (63.7)	30 (70.3)	0.371
Albumin/Globulin, No. (%)			
<1.2	56 (54.9)	48 (47.5)	0.292
1.2–2.4	45 (44.1)	53 (52.5)	
>2.4	1 (1.0)	0 (0.0)	
Lactate dehydrogenase >250 U/L, No. (%)	28 (27.5)	23 (22.8)	0.518
Alkaline phosphatase >125 U/L(Male) & >135 U/L(Female), No. (%)	10 (9.8)	8 (7.9)	0.806
Na+(mmol/L), No. (%)			
<135	20 (19.6)	20 (19.8)	0.764
135–145	79 (77.5)	80 (79.2)	
>145	3 (2.9)	1 (1.0)	
K+(mmol/L), No. (%)			
<3.5	8 (7.8)	19 (18.8)	0.030
3.5–5.5	93 (91.2)	82 (81.2)	
>5.5	1 (1.0)	0 (0.0)	
Anion gap (mmol/L), No. (%)			
<8	18 (17.6)	23 (22.8)	0.099
8–16	80 (78.4)	78 (77.2)	
>16	4 (3.9)	0 (0.0)	
Creatinine ≥97 µmmol/L, No. (%)	28 (27.5)	19 (18.8)	0.183
Urea >8.0 mmol/L, No. (%)	49 (48.0)	42 (41.6)	0.398
FIB(g/L), No. (%)			
<2	3 (2.9)	0 (0.0)	0.204
2–4	44 (43.1)	40 (39.6)	
>4	55 (53.9)	61 (60.4)	
APTT(s), No. (%)			
<24	5 (4.9)	1 (1.0)	0.292
24–40	95 (93.1)	99 (98.0)	
>40	2 (2.0)	1 (1.0)	
TT(s), No. (%)			
<11	0 (0.0)	2 (2.0)	0.495
11–18	101 (99.0)	98 (97.0)	
>18	1 (1.0)	1 (1.0)	
PT1(s), No. (%)			
<9.4	2 (2.0)	0 (0.0)	0.317
9.4–13.8	94 (92.2)	97 (96.0)	
>13.8	6 (5.9)	4 (4.0)	
PTINR, No. (%)			
<0.76	1 (1.0)	0 (0.0)	0.609
0.76–1.16	96 (94.1)	98 (97.0)	
>1.16	5 (4.9)	3 (3.0)	
D-dimmer >500 µg/L, No. (%)	92 (90.2)	98 (97.0)	0.082
CRP >10 mg/L, No. (%)	80 (78.4)	90 (89.1)	0.056
ESR >20 mm/60 min, No. (%)	78 (76.5)	81 (80.2)	0.610
Blood glucose(mmol/L), No. (%)			
<3.9	3 (2.9)	1 (1.0)	0.607
3.9–6.9	81 (79.4)	84 (83.2)	
≥7.0	18 (17.6)	16 (15.8)	
Total bilirubin >23 µmmol/L, No. (%)	18 (17.6)	20 (19.8)	0.722
Ca^2+^ <2.11 mmol/L, No. (%)	57 (55.9)	62 (61.4)	0.477
cl^-^(mmol/L), No. (%)			
<99	18 (17.6)	14 (13.9)	0.516
99–110	76 (74.5)	82 (81.2)	
>110	8 (7.8)	5 (5.0)	
Preoperative hemoglobin (g/L)	104.92 ± 18.28	107.07 ± 19.45	0.419
Postoperative hemoglobin (g/L)	86.82 ± 13.28	89.83 ± 13.62	0.113

*aCCI, age-adjusted Charlson Comorbidity Index; ASA classification, American Society of Anesthesiologists classification; Na^+^, serum sodium; K^+^, serum potassium; Ca2+, serum calcium; cl-, serum chlorine; FIB, fibrinogen; APTT, activated partial thromboplastin time; TT, thrombin time; PT1, prothrombin time 1; PTINR, international normalized ratio of prothrombin time; CRP, C-reactive protein; ESR, erythrocyte sedimentation rate.*

### Prognostic Factors for 1-Year All-Cause Mortality After Hip Arthroplasty in Nonagenarians With Hip Fractures

Table 3 that were based on the univariate and multivariate COX proportional hazards regression analysis presents the predictors for 1-year all-cause mortality in the training set of 102 cases (30 deaths). First, univariate Cox regression analysis was applied to reveal the potential prognostic predictors, and revealed that 11 of 44 predictors were significantly associated with 1-year all-cause mortality. Furthermore, the results of multivariate analysis showed that aCCI (HR = 1.3), ASA classification (III&IV, HR = 2.05), Urea (>8.0 mmol/L, HR = 1.74), serum Ca^2+^(<2.11 mmol/L, HR = 0.54), postoperative hemoglobin (HR = 0.98) were significant predictors for 1-year all-cause mortality after hip arthroplasty in nonagenarians suffered hip fractures.

**Table 3:** Univariate and multivariate COX proportional hazards regression analysis of the fatal outcome of nonagenarians undergoing arthroplasty after hip fracture in the Training cohort.

Variables	Univariate COX analysis		Multivariate COX analysis	
	HR (95% CIs)	*p*	HR (95% CIs)	*p*
Age (≥95 years)	1.04 (0.66–1.66)	0.859		
Gender (female), No. (%)	0.91 (0.55–1.5)	0.698		
Length before admission (hours)				
≤72	Ref.			
73–168	1.08 (0.63–1.83)	0.786		
169–504	1.24 (0.63–2.46)	0.533		
>504	0.62 (0.24–1.62)	0.330		
Preoperative length of stay (days)				
<2	Ref.			
2–3	1.07 (0.54–2.12)	0.837		
>3	1.05 (0.64–1.74)	0.842		
Fracture type (Intertrochanteric fracture)	1.5 (0.93–2.43)	0.097		
Left/Right	1.18 (0.74–1.87)	0.484		
aCCI	1.48 (1.18–1.87)	0.001	1.3 (1.01–1.67)	0.042
Pulse (beats/min)				
<60	Ref.			
60–100	1.29 (0.4–4.11)	0.671		
>100	1.83 (0.47–7.13)	0.384		
Systolic pressure (mmHg)				
90–119	Ref.			
120–139	0.88 (0.39–1.96)	0.750		
140–159	0.77 (0.33–1.8)	0.549		
160–179	1.08 (0.46–2.53)	0.867		
≥180	1.12 (0.33–3.77)	0.856		
Diastolic pressure (mmHg)				
<60	Ref.			
60–79	0.91 (0.36–2.32)	0.840		
80–89	0.94 (0.35–2.48)	0.895		
90–99	0.38 (0.1–1.44)	0.155		
100–109	2.37 (0.27–20.61)	0.434		
Cardiac function classification (NYHA III&IV)	1.26 (0.93–1.7)	0.131		
ASA classification (ASA III&IV)	1.97 (1.22–3.18)	0.006	2.05 (1.22–3.44)	0.007
Operation type (Hemiarthroplasty)	0.88 (0.38–2.07)	0.775		
Anesthesia				
Nerve block anesthesia	Ref.			
Intravertebral anesthesia	1.16 (0.52–2.58	0.714		
General anesthesia	1.01 (0.36–2.78	0.989		
Operation time (min)				
<60	Ref.			
60–120	0.88 (0.35–2.21)	0.791		
>120	0.98 (0.32–3.03)	0.978		
Blood transfusion	1.51 (0.86–2.64)	0.148		
White-cell count (×10^9^/L)				
<4.00	Ref.			
4.00–10.00	1.3 (0.3–5.72)	0.728		
≥10.01	1.82 (0.39–8.44)	0.442		
Platelet count (×10^9^/L)				
<100	Ref.			
100–300	1.14 (0.54–2.4)	0.724		
>300	1.48 (0.53–4.12)	0.451		
Percentage of neutrophl >75%	1.94 (1.2–3.13)	0.007		
Hematocrit <40 (Male), <35 (Female)	1.72 (0.92–3.21)	0.087		
Albumin <35 g/L	0.81 (0.49–1.32)	0.392		
Albumin/Globulin				
<1.2	Ref.			
1.2–2.4	0.58 (0.36–0.95)	0.029		
>2.4	0.9 (0.12–6.56)	0.916		
Lactate dehydrogenase >250 U/L	1.12 (0.67–1.86)	0.660		
Alkaline phosphatase >125 U/L (Male) & >135 U/L (Female)	1.06 (0.51–2.21)	0.877		
Na+(mmol/L)				
<135	Ref.			
135–145	0.72 (0.4–1.3)	0.270		
>145	13.36 (3.36–53.21)	<0.001		
K+(mmol/L)				
<3.5	Ref.			
3.5–5.5	0.52 (0.25–1.1)	0.085		
>5.5	7.89 (0.92–67.77)	0.060		
Anion gap (mmol/L)				
<8	Ref.			
8–16	0.95 (0.51–1.78)	0.879		
>16	1.93 (0.62–6.07)	0.258		
Creatinine ≥97 µmmol/L	1.86 (1.14–3.04)	0.014		
Urea >8.0 mmol/L	2.02 (1.25–3.26)	0.004	1.74 (1.06–2.85)	0.028
FIB (g/L)				
<2	Ref.			
2–4	0.94 (0.27–3.19)	0.915		
>4	1.28 (0.38–4.29)	0.686		
APTT(s)				
<24	Ref.			
24–40	0.36 (0.14–0.92)	0.032		
>40	3 (0.56–16.03)	0.199		
TT(s)	3.29 (0.45–24.21)	0.242		
PT1(s)				
<9.4	Ref.			
9.4–13.8	1.05	0.946		
>13.8	0.9	0.896		
PTINR				
<0.76	Ref.			
0.76–1.16	1.87 (0.26–13.54)	0.537		
>1.16	0.79 (0.07–8.7)	0.844		
D-dimmer >500 µg/L	1.7 (0.73–3.95)	0.218		
CRP >10 mg/L	1.44 (0.81–2.54)	0.212		
ESR >20 mm/60 min	1.04 (0.61–1.78)	0.889		
Blood glucose (mmol/L)				
<3.9	Ref.			
3.9–6.9	0.56 (0.17–1.81)	0.334		
≥7.0	0.71 (0.2–2.48)	0.590		
Total bilirubin >23 µmmol/L	1.52 (0.84–2.73)	0.163		
Ca^2+^ <2.11 mmol/L	0.6 (0.37–0.95)	0.030	0.54 (0.33–0.87)	0.011
cl^-^(mmol/L)				
<99	Ref.			
99–110	1.01 (0.54–1.89)	0.977		
>110	1.49 (0.56–3.98)	0.424		
Preoperative hemoglobin (g/L)	0.99 (0.98–1)	0.044		
Postoperative hemoglobin (g/L)	0.97 (0.96–0.99)	0.003	0.98 (0.96–1.00)	0.024

*aCCI, age-adjusted Charlson Comorbidity Index; ASA classification, American Society of Anesthesiologists classification; Na^+^, serum sodium; K^+^, serum potassium; Ca^2+^, serum calcium; cl-, serum chlorine; FIB, fibrinogen; APTT, activated partial thromboplastin time; TT, thrombin time; PT1, prothrombin time 1; PTINR, international normalized ratio of prothrombin time; CRP, C-reactive protein; ESR, erythrocyte sedimentation rate.*

### Development and Validation of the Prognostic Nomogram

**Figure 1:**
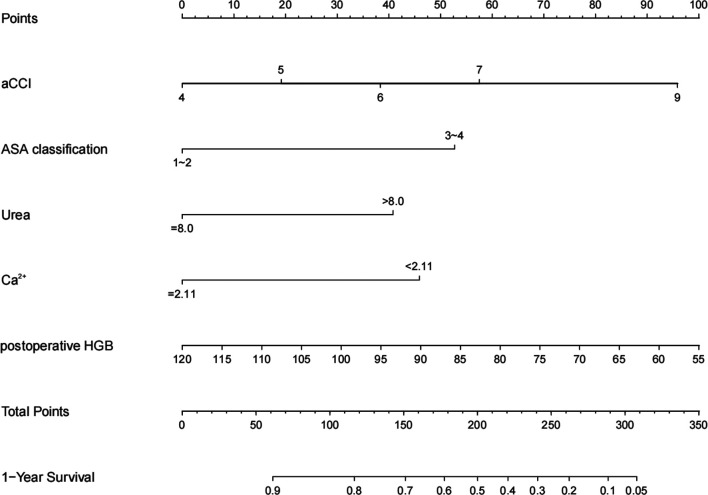
A nomogram for prediction of 1-year mortality after arthroplasty in nonagenarians with hip fractures. aCCI, age-adjusted Charlson Comorbidity Index; ASA classification, American Society of Anesthesiologists classification; Ca^2+^, serum calcium; HGB, hemoglobin.

**Figure 2:**
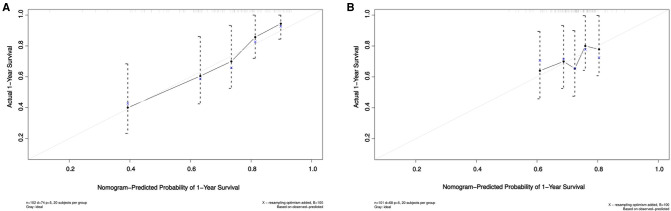
Calibration curves. (**A**) Calibration curves showing the probability of 1-year mortality between the nomogram prediction and the actual observation in training set; (**B**) Calibration curves showing the probability of 1-year mortality between the nomogram prediction and the actual observation in the validation set. The prediction probability of the nomogram for 1-year mortality was plotted on the X-axis, and the actual probability was plotted on the Y-axis**.**

Based on the Akaike information criteria (AIC), 5 independent prognostic predictors, including aCCI (HR = 1.3, 95% CI, 1.01–1.67, *p *= 0.042), ASA classification (HR = 2.05, 95% CI, 1.22–3.44, *p *= 0.007), Urea (HR = 1.74, 95% CI, 1.06–2.85, *p *= 0.028), serum Ca^2+^ (HR = 0.54, 95% CI, 0.33–0.87, *p *= 0.011), postoperative hemoglobin (HR = 0.98, 95% CI, 0.96–1.00, *p *= 0.024) were selected for the construction of the prognostic nomogram (Figure 1). The C-index for 1-year all-cause mortality after hip arthroplasty in nonagenarians with hip fractures was 0.71 (95% CIs, 0.68–0.78). The optimal agreement between nomogram-predicted probability and the result of observation in both the training and validation set was showed clearly by the calibration curve (Figure 2).

## Discussion

One-year mortality after hip fractures surgery in the elderly is more than 27% (10–13). In our study, the one-year mortality is as high as 29.6% in nonagenarians. These extremely old patients have high operative risk and difficult perioperative management and require more precise treatment. Risk prediction is one of the advantages of precision medicine, and the precision of prediction models need to develop from cohort customization to personalize management. Accompanied by the development of artificial intelligence, new risk prediction models are emerging constantly, we applied Cox regression models and predictive nomogram in the current study, aimed to develop a simple and intuitive model to interpret complex clinical indicators, to help clinicians screen out high-risk patients, and to help patients understand the high mortality associated with these factors easily. To our knowledge, this is the first study to use one new nomogram model to predict the risk factors for mortality after arthroplasty for nonagenarians with hip fractures. In the past, most relevant studies had focused on the incidence of adverse events during the postoperative hospital stay or one month after surgery, while we followed the cohort for up to two decades, and the focus of the study was not limited to short-term survival rate but extended to medium and long-term survival rate. We limited the subjects to over 90 years old and arthroplasty for hip fractures, so as to reduce the influence of bias and confounding factors as much as possible. A stable predictive nomogram was constructed by COX regression, and aCCI, ASA classification, Urea, serum Ca2+, and postoperative hemoglobin were confirmed as the potential independent risk factors for 1-year all-cause mortality after hip arthroplasty in nonagenarians with hip fractures.

Comorbidities are common in the elderly with hip fractures. Patients with three or more comorbidities or a complex medical history have significantly increased mortality compared with those with no comorbidities (14, 15). Multiple comorbidities often co-exist and interact with each other. It is difficult to summarize the overall organ or body function by analyzing a single or specific type of disease. In order to comprehensively evaluate and quantify patients’ comorbidities and physiological functions, aCCI and ASA classification were introduced in our study. aCCI is an assessment tool that combines age factors and quantifies a variety of comorbidities. aCCI and Charlson comorbidity index (CCI) are both predictors of post-operative complications with aCCI being the better predictive determinant (16). The ASA classification includes five levels to assess the patient’s physical condition and perioperative risk. Both aCCI and ASA classification are common tools for operative risk stratification. Previous studies have attempted to use CCI and ASA for risk stratification and suggested that CCI performed better than ASA classification in the prediction for 1-year mortality after surgery for hip fractures (17, 18). However, some studies suggested that ASA classification was an independent risk factor for it, rather than CCI (19). In our study, both aCCI and ASA classification are potential risk factors, which consists of the report of Ek et al. (20). Additionally, we realized that serum urea, as one of the indicators of renal function, is an independent risk factor for postoperative mortality. This may indicate that renal disease as a serious comorbidity has a great impact on prognosis.

Hypocalcemia occurs in more than 30% of elderly orthopedic patients (21, 22). Clinical symptoms related to it are rare, but calcium ion is an important electrolyte, neurotransmitter and coagulation factor, so perioperative hypocalcemia is closely related to blood loss (21) and delirium (23). Even more, chronic hypocalcemia can severely affect cardiac contractility and electrical activity (24), which undoubtedly increases the risk of perioperative adverse events. Xu et al. (25) found that hypocalcemia could shorten the overall survival of the elderly, and our study regarded hypocalcemia as a potential risk factor for mortality within 1 year after surgery. Chewcharat et al. (26) found that the fluctuating serum sodium trajectory and hyponatremia was associated with the high in-hospital and 1-year mortality. Hypokalemi is common in hypertensive patients with hypotensor treatment and was considered to be associated with increased all-cause and cardiovascular mortality (27). While our study did not find the potential association between hypernatremia, hypokalemia and postoperative mortality, this may be due to the limitations of sample size or the complexity of the physiology of nonagenarians or differences in time periods. We should pay more attention to hypocalcemia and other blood indexes that may increase the mortality risk, and addressed them preoperatively for optimization of patient status for the operation.

Anemia is one of the most common complications after surgery for hip fracture in the elderly and is also an important factor leading to delirium, weakness, poor recovery of daily life, and even death (28, 29). Anemia on hospital admission is an independent predictor for long-term mortality in patients with hip fractures (30). During the perioperative period, hemoglobin is a direct indicator for the identification of anemia and the main basis for blood transfusion (31–34), meanwhile it is helpful for the classification of anemia and guiding perioperative fluid management in combination with hematocrit. Durán-Nah et al. (35) reported that intraoperative bleeding ≥400 mL and preoperative hemoglobin <11 g/dL were closely related to allogeneic blood transfusion. In our study, we included not only preoperative hemoglobin but also postoperative hemoglobin to facilitate dynamic assessment of its risk. Moreover, we found that postoperative hemoglobin is more meaningful than preoperative hemoglobin, which generally reflects the body’s ability to recover from the shock of surgery. Our experience in treatment of anemia is restrictive blood transfusion: when hemoglobin is lower than 80 g/L, red blood cell is given; anemia should be corrected first before operation, and hemoglobin should be tested dynamically after operation to determine the timing of blood transfusion.

There were several limitations in this study. First, be subjected to the limitations of description bias of medical records and the great time span, some information that may affect prognosis was not analyzed in detail, such as pre- and postoperative cognitive function (cognitive deficit, dementia), preoperative activity of daily living, including habitual physical activity level, preoperative residence, preoperative physical function (e.g., handgrip strength, mobility, activity of daily living index), postacute rehabilitation of the patients. Second, it is a single-center, retrospective study with relatively small sample size, which was insufficient to generate a model containing all potential confounding factors and lacked external validation. Third, the development and validation sets were affected by selection bias. Besides, due to the low mortality and small number of deaths during the period from postoperative hospitalization to six months in our study, we did not analyze the risk factors for mortality at different time points during this period.

## Conclusion

We established and validated a novel nomogram based on aCCI, ASA classification, Urea, serum Ca^2+^ and postoperative hemoglobin, that could effectively predict 1-year all-cause mortality after hip arthroplasty in nonagenarians with hip fracture. It would be helpful for clinicians to identify high-risk patients, conduct reasonable and individualized perioperative managements.

## Data Availability

The raw data supporting the conclusions of this article will be made available by the authors, without undue reservation.

## References

[1] Fang EF Xie C Schenkel JA Wu C Long Q Cui H A research agenda for ageing in China in the 21st century (2nd edition): focusing on basic and translational research, long-term care, policy and social networks. Ageing Res Rev. (2020) 64:101174. 10.1016/j.arr.2020.101174 32971255 PMC7505078

[2] United Nations, Department of Economic and Social Affairs, Population Division. World population ageing 2017 (ST/ESA/SER.A/408). (2017). Available from: https://population.un.org/ProfilesOfAgeing2017/index.html (Accessed October 15, 2018).

[3] Zhang C Feng J Wang S Gao P Xu L Zhu J Incidence of and trends in hip fracture among adults in urban China: a nationwide retrospective cohort study. PLoS Med. (2020) 17(8):e1003180. 10.1371/journal.pmed.1003180 32760065 PMC7410202

[4] Bhandari M Swiontkowski M. Management of acute hip fracture. N Engl J Med. (2017) 377(21):2053–62. 10.1056/NEJMcp1611090 29166235

[5] Tu DP Liu Z Yu YK Xu C Shi XL. Internal fixation versus hemiarthroplasty in the treatment of unstable intertrochanteric fractures in the elderly: a systematic review and meta-analysis. Orthop Surg. (2020) 12(4):1053–64. 10.1111/os.12736 32691520 PMC7454150

[6] Holt G Macdonald D Fraser M Reece AT. Outcome after surgery for fracture of the hip in patients aged over 95 years. J Bone Joint Surg Br. (2006) 88(8):1060–4. 10.1302/0301-620X.88B8.17398 16877606

[7] Bokshan SL Marcaccio SE Blood TD Hayda RA. Factors influencing survival following hip fracture among octogenarians and nonagenarians in the United States. Injury. (2018) 49(3):685–90. 10.1016/j.injury.2018.02.004 29426609

[8] Mayordomo-Cava J Abásolo L Montero-Fernandez N Ortiz-Alonso J Vidán-Astiz M Serra-Rexach JA. Hip fracture in nonagenarians: characteristics and factors related to 30-day mortality in 1177 patients. J Arthroplasty. (2020) 35(5):1186–93. 10.1016/j.arth.2019.12.044 31992530

[9] Leal JA Garcia LF Peña OR Gomez-Gelvez A. Patients aged ninety years and older are exposed to increased risk of one-year mortality after hip fractures. Eur J Orthop Surg Traumatol. (2021) 31(7):1501–6. 10.1007/s00590-021-02918-0 33651223

[10] NHFD. *Annual Report*. (2019). Available from: https://www.nhfd.co.uk/20/hipfractureR.nsf/docs/2019Report (Accessed 18, 2020).

[11] Gundel O Thygesen LC Gögenur I Ekeloef S. Postoperative mortality after a hip fracture over a 15-year period in Denmark: a national register study. Acta Orthop. (2020) 91(1):58–62. 10.1080/17453674.2019.1680485 31635502 PMC7006693

[12] McLeod G Kennedy I Simpson E Joss J Goldmann K. Pilot project for a web-based dynamic nomogram to predict survival 1 year after hip fracture surgery: retrospective observational study. Interact J Med Res. (2022) 11(1):e34096. 10.2196/34096 35238320 PMC9008534

[13] Forssten MP Bass GA Ismail AM Mohseni S Cao Y. Predicting 1-year mortality after hip fracture surgery: an evaluation of multiple machine learning approaches. J Pers Med. (2021) 11(8):727. 10.3390/jpm11080727 PMC8401745 34442370

[14] Roche JJ Wenn RT Sahota O Moran CG. Effect of comorbidities and postoperative complications on mortality after hip fracture in elderly people: prospective observational cohort study. BMJ. (2005) 331(7529):1374. 10.1136/bmj.38643.663843.55 16299013 PMC1309645

[15] Crawford ZT Southam B Matar R Avilucea FR Bowers K Altaye M A nomogram for predicting 30-day mortality in elderly patients undergoing hemiarthroplasty for femoral neck fractures. Geriatr Orthop Surg Rehabil. (2020) 11:2151459320960087. 10.1177/2151459320960087 33117596 PMC7573749

[16] Marya SK Amit P Singh C. Impact of Charlson indices and comorbid conditions on complication risk in bilateral simultaneous total knee arthroplasty. Knee. (2016) 23(6):955–9. 10.1016/j.knee.2016.05.013 27802921

[17] Pan L Ning T Wu H Liu H Wang H Li X Prognostic nomogram for risk of mortality after hip fracture surgery in geriatrics. Injury. (2022) 53(4):1484–9. 10.1016/j.injury.2022.01.029 35078620

[18] Varady NH Gillinov SM Yeung CM Rudisill SS Chen AF. The Charlson and elixhauser scores outperform the american society of anesthesiologists score in assessing 1-year mortality risk after hip fracture surgery. Clin Orthop Relat Res. (2021) 479(9):1970–9. 10.1097/CORR.0000000000001772 33930000 PMC8373577

[19] Quach LH Jayamaha S Whitehouse SL Crawford R Pulle CR Bell JJ. Comparison of the Charlson Comorbidity Index with the ASA score for predicting 12-month mortality in acute hip fracture. Injury. (2020) 51(4):1004–10. 10.1016/j.injury.2020.02.074 32151423

[20] Ek S Meyer AC Hedström M Modig K. Comorbidity and the association with 1-year mortality in hip fracture patients: can the ASA score and the Charlson Comorbidity Index be used interchangeably? Aging Clin Exp Res. (2022) 34(1):129–36. 10.1007/s40520-021-01896-x 34106421 PMC8795011

[21] Wang Z Chen X Chen Y Yang L Wang H Jiang W Low serum calcium is associated with perioperative blood loss and transfusion rate in elderly patients with hip fracture: a retrospective study. BMC Musculoskelet Disord. (2021) 22(1):1025. 10.1186/s12891-021-04914-1 34876077 PMC8653606

[22] Gai P Sun H Sui L Wang G. Hypocalcaemia after total knee arthroplasty and its clinical significance. Anticancer Res. (2016) 36(3):1309–11. 26977030

[23] Wang LH Xu DJ Wei XJ Chang HT Xu GH. Electrolyte disorders and aging: risk factors for delirium in patients undergoing orthopedic surgeries. BMC Psychiatry. (2016) 16(1):418. 10.1186/s12888-016-1130-0 27881118 PMC5120472

[24] Yang YC Shen FR Lu YQ. Hypocalcemia: a reversible cause of T wave alternans and heart failure. J Zhejiang Univ Sci B. (2014) 15(6):598–600. 10.1631/jzus.B1400078 24903998 PMC4082537

[25] Xu J Chen X Wang X Zhu C Hu Y Yang X Preoperative hyponatremia and hypocalcemia predict poor prognosis in elderly gastric cancer patients. Cancer Manag Res. (2019) 11:8765–80. 10.2147/CMAR.S211603 31632136 PMC6775496

[26] Chewcharat A Thongprayoon C Cheungpasitporn W Mao MA Thirunavukkarasu S Kashani KB. Trajectories of serum sodium on in-hospital and 1-year survival among hospitalized patients. Clin J Am Soc Nephrol. (2020) 15(5):600–7. 10.2215/CJN.12281019 32213501 PMC7269204

[27] Krogager ML Søgaard P Torp-Pedersen C Bøggild H Lee CJ Bonde A Impact of plasma potassium normalization on short-term mortality in patients with hypertension and hypokalemia or low normal potassium. BMC Cardiovasc Disord. (2020) 20(1):386. 10.1186/s12872-020-01654-3 32838735 PMC7446172

[28] Gregersen M. Postoperative red blood cell transfusion strategy in frail anemic elderly with hip fracture. A randomized controlled trial. Dan Med J. (2016) 63(4):B5221. 27034188

[29] Doody K Mohamed KM Butler A Street J Lenehan B. Adverse event recording post hip fracture surgery. Ir Med J. (2013) 106(10):300–2. 24579408

[30] Zhang L Yin P Lv H Long A Gao Y Zhang L Anemia on admission is an independent predictor of long-term mortality in hip fracture population: a prospective study with 2-year follow-up. Medicine (Baltimore). (2016) 95(5):e2469. 10.1097/MD.0000000000002469 26844456 PMC4748873

[31] Kim MS Kim JD Ro KH Park JJ Rhee YG. Hematologic profile in reverse total shoulder arthroplasty: perioperative and postoperative blood loss. J Shoulder Elbow Surg. (2019) 28(9):1737–42. 10.1016/j.jse.2019.01.027 30981547

[32] Fazal MA Bagley C Garg P. Predictors for perioperative blood transfusion in elderly patients with extra capsular hip fractures treated with cephalo-medullary nailing. Chin J Traumatol. (2018) 21(1):16–9. 10.1016/j.cjtee.2017.09.002 29398291 PMC5857894

[33] Luangwaranyoo A Suksintharanon M Tangadulrat P Iamthanaporn K Hongnaparak T Yuenyongviwat V. Factors for blood transfusions following hemi hip arthroplasty for patients with femoral neck fracture. Geriatr Orthop Surg Rehabil. (2020) 11:2151459320972993. 10.1177/2151459320972993 33282448 PMC7686587

[34] Wang JQ Chen LY Jiang BJ Zhao YM. Development of a nomogram for predicting blood transfusion risk after hemiarthroplasty for femoral neck fractures in elderly patients. Med Sci Monit. (2020) 26:e920255. 10.12659/MSM.920255 32074099 PMC7043352

[35] Durán-Nah JJ Pastelín-Ruiz SE Miam-Viana Ede J. Perioperative risk factors associated with allogeneic blood transfusion in patients with hip fracture surgery. Rev Med Inst Mex Seguro Soc. (2015) 53(4):406–13. 26177427

